# RETRACTION: Comparative Outcomes of Autologous Cultured Melanocytes Transplantation and Non‐Cultured Epidermal Cell Suspension Transplantation in Piebaldism Patients: A Retrospective Study

**DOI:** 10.1111/srt.70226

**Published:** 2025-09-18

Jin R, Hong W, Ye Z, Fu L, Hu W, Xu A. Comparative outcomes of autologous cultured melanocytes transplantation and non‐cultured epidermal cell suspension transplantation in piebaldism patients: A retrospective study. *Skin Res Technol*. 2024;30(1):e13580. doi:10.1111/srt.13580

The above article, published online on 15 January 2024 in Wiley Online Library (wileyonlinelibrary.com), and has been issued by agreement between Skin Research and Technology; and John Wiley & Sons Ltd. The article was accepted for publication following an insufficient peer review process that does not meet the standards of the journal's peer review policy. Furthermore, scientific flaws have been identified that affect the validity of the conclusions drawn. Accordingly, the article has been retracted. The authors have been informed of this decision but were unavailable for final confirmation.

